# Enhancing Mechanical and Electrochemical Stability of EDLC Electrodes via Crosslinked Polysaccharide Binder Blends

**DOI:** 10.1002/advs.202520621

**Published:** 2025-12-23

**Authors:** Mahdi Karimi Jafari, Rupesh Singh, Stefano Passerini, Alberto Varzi

**Affiliations:** ^1^ Karlsruhe Institute of Technology (KIT) Karlsruhe Germany; ^2^ Helmholtz Institute Ulm (HIU) Ulm Germany; ^3^ Austrian Institute of Technology (AIT) Center of Transport Technologies Vienna Austria

**Keywords:** crosslinking, electric double‐layer capacitors (EDLCs), polysaccharides, sustainable electrodes, water processable binders

## Abstract

Advancing sustainable energy storage technologies has increased the focus on water processable binders for high‐performance battery and capacitor electrodes. In this work, a blend of potato starch (PS) and xanthan gum (XG) is chemically cross‐linked with four carboxylic acids (citric, malic, succinic, and glutaric) to improve the mechanical and electrochemical performance of the electric double‐layer capacitors (EDLCs) electrodes. Low‐temperature cross‐linking via esterification is confirmed by FTIR and TGA analysis, showing ester bond formation already at 80 °C. Mechanical characterization demonstrates that cross‐linked binders, particularly citric acid (PXC) and malic acid (PXM), significantly improve adhesion strength (up to 0.38 MPa for PXC) and reduce elastic deformation, correlating with increased cross‐linking density. Electrochemical tests demonstrate that PXC and PXM electrodes achieve superior capacitance retention at high current densities and long‐term floating voltage conditions. After 500 h at 3 V, cross‐linked electrodes retain over 91% of their initial capacitance, compared to 84% for the non‐cross‐linked reference. Electrochemical impedance spectroscopy further shows that cross‐linked binders display notably lower contact and interfacial resistances, even after long term floating test. This study highlights the potential of cross‐linked polysaccharide binders as a promising green binder system to enhance mechanical integrity and long‐term high voltage electrochemical stability of EDLC electrodes.

## Graphical abstract

Cross‐linked polysaccharide binders derived from potato starch and xanthan gum enable fully water processed EDLC electrodes with improved mechanical integrity and long‐term electrochemical stability at high voltage.

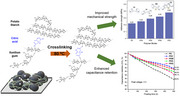

## 1 Introduction

Electric Double Layer Capacitors (EDLCs) store energy simply via the formation of an electric double‐layer at the electrode/electrolyte interface (capacitive storage). Since no faradaic (redox) reaction or chemical modification of the electrode occurs, EDLCs can display excellent cycle life (up to millions of cycles), fast charge/discharge rates, and subsequently very high‐power density (>10 kW kg^−1^) [1, 2].

EDLCs’ performance heavily depends on the choice of electrolyte and active electrode materials (e.g., activated carbon). However, inactive electrode components play a crucial role as well. For example, despite its limited content in the electrode, the binder plays a crucial role in maintaining electrode integrity and overall electrochemical stability [3]. Conventionally, fluorinated thermoplastic polymers such as polytetrafluoroethylene (PTFE) and polyvinylidene difluoride (PVDF) have been widely used as binders in both batteries and capacitors [4, 5]. However, the presence of fluorine and the need of toxic organic solvents like N‐methyl‐2‐pyrrolidone (NMP) for slurry preparation raise significant environmental and safety concerns [6]. To address these issues, fluorinated binders are progressively being replaced with more sustainable and water‐processable polymers like polyvinylpyrrolidone (PVP) [7], carboxymethylcellulose (CMC) [8], casein [9], natural cellulose [10], etc. These polymer binders offer multiple advantages, such as low cost, environmental friendliness, and safer manufacturing processes. Among water‐processable polymers, polysaccharides have received particular interest due to their natural abundance, biodegradability, and ability to form strong adhesive networks. In recent years, various polysaccharides, including starch [11, 12] and xanthan gum [13], have been proposed due to abundant hydroxyl groups, which promote strong adhesion and stability. These were investigated as eco‐friendly alternative binders not only in EDLCs and batteries but in other application fields as well, like food packaging and pharmaceutical formulations [14, 15, 16, 17, 18].

Despite the promising advantages of polysaccharide‐based binders, their implementation in EDLC electrodes at the industrial level is still challenging. One of the primary difficulties is maintaining electrode integrity, particularly in high mass‐loading electrodes. For instance, carboxymethylcellulose (CMC) often shows cracking during the drying process, making it difficult to achieve uniform and mechanically stable electrodes [11, 12]. The addition of a rubberizing agent such as SBR (styrene‐butadiene rubber) has proven very effective to mitigate this issue [19, 20]. However, SBR is a petroleum‐derived synthetic polymer with poor bio‐degradability and should ideally be avoided [21]. Therefore, alternative binder formulations are urgently needed.

Starch is a cost‐effective and high‐viscosity polysaccharide rich in hydroxyl groups, which promote strong adhesion and stability [22]. Its abundant hydroxyl groups give it excellent film‐forming properties and strong intermolecular cohesion, essential for binder performance at high mass loadings [12]. Blending starch with other polysaccharides such as xanthan gum has been reported to improve the mechanical and functional properties of starch‐based materials in the food industry, due to enhanced hydrogen bonding and network formation between the two polymers [18, 23]. Xanthan gum, with its rigid helical backbone and pronounced shear‐thinning behavior, stabilizes the slurry against particle sedimentation and ensures uniform electrode coatings [18]. The combination of starch and xanthan gum enables the formation of an interpolymer network through increased hydrogen bonding and network formation between the two polymers [16, 17, 23].

Another effective strategy to further improve the performance of polysaccharide polymers is chemical cross‐linking via esterification reaction, which involves the formation of covalent ester bonds between carboxyl (─COOH) and hydroxyl (─OH) functional groups through a thermal condensation reaction. The resulting cross‐linked polymer network provides enhanced mechanical strength, flexibility, and durability, as demonstrated in various applications [24, 25, 26]. Citric acid (CA) is commonly used to cross‐link polysaccharides. For instance, cross‐linking with CA enhanced the mechanical strength and thermal stability of starch‐based films [26]. Similar studies have shown that cross‐linking polysaccharides such as chitosan and starch with CA at different concentrations can improve film performance. On the other hand, an excessive amount of cross‐linking agent may negatively impact the film properties due to acid‐induced hydrolysis of the polysaccharide chains. This leads to a disruption of the binder network, which reduces mechanical strength and cross‐linking efficiency [24].

Several studies have also highlighted the benefits of cross‐linked polysaccharide binders in energy storage applications. In silicon‐based electrodes for Li‐ion batteries, corn starch cross‐linked with maleic anhydride has been reported to enhance electrode durability [14]. Similarly, Kuenzel et al. reported that CMC and guar gum (GG) binders cross‐linked with CA can improve the mechanical stability of LiNi_0.5_Mn_1.5_O_4_ (LNMO)‐based electrodes and rate capability of Li‐ion cells [27]. Furthermore, Bargnesi et al. demonstrated how chitosan cross‐linked with succinic acid enabled stable and flexible freestanding electrodes (i.e., without a current collector) for Na‐ion cells [28].

The selection of the right cross‐linking agent plays an important role in the final properties of a cross‐linked polymer, as it may influence the network structure, chain interactions, and overall cross‐linking behavior of polymers [26, 28, 29]. For instance, citric acid (CA) [27], D, L‐Malic acid (MA) [30], glutaric acid (GA) [29], and succinic acid (SA) [28] are carboxylic acids commonly used as cross‐linking agents. CA is a tri‐carboxylic acid and forms strong cross‐linking through esterification reaction, usually when heated up to 150°C. MA is a dicarboxylic acid with an additional hydroxyl (‐OH) group, which could also provide improved adhesion and flexibility through cross‐linking reaction. GA and SA are both dicarboxylic acids, and cross‐linking occurs through esterification as well, but their different chain lengths may impact the properties of the binder network. SA exhibits shorter chain length in a more rigid structure, whereas GA displays longer chain length and potentially enhanced flexibility of the cross‐linked network. The structural and chemical differences among these cross‐linking agents (CA, MA, GA, and SA) may directly influence relevant properties such as adhesion, elasticity, and overall electrode performance.

While multiple reports exist on cross‐linked polysaccharide binders in both Li‐ion and Na‐ion batteries (e.g., corn starch cross‐linked with maleic anhydride [14]; CA‐crosslinked CMC and guar gum [27]; succinic acid‐crosslinked chitosan [28]), such studies primarily focus on demonstrating performance improvements, rather than correlating the electrodes' properties with the chemical nature of the cross‐linked binder network. The role of different carboxylic acids as cross‐linking agents has been reported in other fields. For example, Gebresas et al. systematically studied how citric, tartaric, and oxalic acid affect adhesion, flexibility, and network density of starch‐based films for non‐food applications [31]. However, to the best of the authors’ knowledge, there is no established understanding on the relationship between cross‐linker chemistry and properties relevant for electrochemical energy storage devices, such as adhesion to metal foils, electrochemical and mechanical stability, and ability to obtain thick high‐loading electrodes.

In this work, we comprehensively study the effect of CA, MA, SA, and GA as cross‐linking agents (CLA) in a water‐based polymer binder blend of potato starch and xanthan gum. These four cross‐linkers were carefully selected with the aim of correlating key chemical features (number of carboxylic and hydroxylic groups, length of the alkylic chain) and EDLC electrode properties. By means of multiple characterization techniques such as Fourier‐transform infrared spectroscopy (FTIR), thermogravimetric analysis (TGA), scanning electron microscopy (SEM), adhesion tape test, and various electrochemical methods (including EIS), we demonstrate how the cross‐linker chemistry can largely influence the electrochemical performance of EDLCs.

## 2 Materials and Methods

### 2.1 Materials

The materials used in this work were procured from various sources. Potato Starch (PS, SIALS2004), Citric acid (CA, 99%, C0759), Glutaric acid (GA, 99%, 800295), and Succinic acid (SA, 99%, 8222600250) were purchased at Sigma‐Aldrich, while DL‐Malic acid (MA, 99%, ACRO12525) at Thermo Fisher Scientific. Xanthan gum (XG) was instead provided by Lamberti Spa, and Carbon Black (C‐ENERGY Super C45) by Imerys Graphite & Carbon. Skeleton Technologies GmbH generously supplied Curved Graphene (CG) active material powder, rubberizing agent (in the form of aqueous suspension with 40 wt.% solid content), separators, and etched aluminum current collector foils (with a thickness of around 20 µm). Since most of these materials are proprietary to Skeleton Technologies GmbH and are covered by confidentiality agreements, no further information can be disclosed about their chemical, structural, and morphological properties. Some non‐sensitive information about the CG active material (used in all electrodes in this work) can be found in our earlier joint publication with Skeleton Technologies GmbH [32]. The 1 m dimethylpyrrolidinium tetrafluoroborate (Pyr_11_BF_4_) in acetonitrile (ACN) solution used as electrolyte was generously supplied by E‐Lyte.

### 2.2 Preparation and Characterization of Polymer Binder Blends

In order to study the cross‐linking process and its influence on the properties of the polymer blends, 2 wt.% binder solutions were first prepared. The 2% binder solid content included both the PS and XG polymers and the CLA (when added). PS and XG were used in a weight ratio of 3:1. This polymer blend was then cross‐linked with different cross‐linking agents (namely CA, GA, MA, and SA), in a weight ratio of 9:1 (blend: CLA). The specific weight compositions of the prepared binders are detailed in Table S1. The preparation process begins with the dissolution of PS in milliQ water. This is achieved by stirring the mixture with a magnetic bar at 500 rpm at room temperature for 3 h. In the case of cross‐linking, an appropriate amount of CLA is first dissolved in milliQ water, after which PS is added. Following this, a specific amount of XG is introduced into the solution and stirred under the same conditions for 24 h. To further examine the properties of these binder blends, the solutions were cast into films. The polymer solutions were poured into glass Petri dishes and left to dry overnight in an oven set at 80°C. The occurrence of cross‐linking via esterification reaction between PS and XG with the cross‐linking agents was further studied using Fourier‐transform infrared spectrometry (FTIR, spectrometer Spectrum Two PerkinElmer) in the range 4000 – 400 cm^−1^, with 32 scans per spectrum. and thermogravimetric analysis (TGA, using a Netzsch STA 409 PC) by heating ca. 4 mg of sample from 40°C to 700°C with a heating rate of 10°C min^−1^ under N_2_ atmosphere.

### 2.3 Preparation and Mechanical Characterization of EDLC Electrodes

The fabrication of water‐based EDLC electrodes involves two key steps. First, a primer layer is applied to the current collector. The slurry for the primer layer was prepared by mixing a 2 wt.% binder solution (see previous section about composition and preparation details) and Carbon Black (Super C45, Imerys Graphite & Carbon). The binder: Carbon Black weight ratio was 1:1. The mixture was homogenized at 2000 rpm for 4 min by a Thinky mixer (Model ARE 250). This slurry was then applied to an etched Al foil current collector using an adjustable doctor blade, with a wet thickness of 30 µm at a coating speed of 50 mm s^−1^. The layer was then dried in an 80°C oven for 20 min. Afterward, the active layer was applied. The solid content of the active layer slurry consisted of 91% Curved Graphene (CG, provided by Skeleton) active material, 3% Carbon Black, 2% rubberizing agent, and 4% binders (again, starting from a 2 wt.% solution). The rubberizing agent was added to ensure sufficient electrode bendability and avoid delamination, especially at high mass loadings (a function analogous to that of SBR in CMC‐based electrodes [19]). A controlled amount of water was added to the slurry to adjust the solid content to 27%. Such slurry was mixed at 2000 rpm for 7 min by a Thinky mixer, then coated onto the pre‐dried primer layer, again using an adjustable doctor blade. This time, however, a wet thickness of 200 µm was set at a coating speed of 50 mm s^−1^. Following the slurry application, the electrodes were first allowed to dry at room temperature for 1 h, and then further dried in an 80°C oven overnight. The final dry thickness of the prepared electrodes (including the active material layer, primer layer, and aluminum current collector) was approximately 105 µm, with active materials mass loading of approximately 6 mg cm^−2^ and a primer layer mass (carbon+binder) loading of approximately 1 mg cm^−2^, excluding current collector weight. Finally, the prepared electrodes were gently calendared using a laboratory calendar at room temperature using an electric rolling press (MSK‐HRP‐01, MTI Corporation) with a roller width of 100 mm at a constant rolling speed (5 mm s^−1^) to improve cohesion and adhesion properties and ensure optimal performance [33]. It should be noted that the binder composition used in the primer layer was always the same as the active layer in all prepared electrodes. The main reason to have a primer layer is to improve the mechanical properties of the electrodes (adhesion and cohesion), as already documented in former studies [34, 35].

To investigate the morphological features of the electrodes, SEM images of both the electrodes’ surface and their cross‐sections were collected by using a field emission scanning electron microscope (ZEISS Crossbeam XB340) equipped with a dual‐beam focused ion beam (FIB) operated at an acceleration voltage of 5 kV. Cross‐section samples were prepared in a Capella FIB system with a gallium ion source, applying a milling current of 3 nA. Cross‐sectional SEM imaging following FIB preparation was performed with the same instrument. Tilt correction using SmartSEM software was applied in order to compensate for image distortion due to the normal 54° stage tilt relative to the optical axis.

The elastic deformation of the electrodes (i.e., the reversible deformation under applied stress) was determined by a compression test performed with a ZwickiLine 2.5 kN (ZwickRoell) with a flat tip with a diameter of 5 mm^2^ at a speed of 0.5 mm min^−1^. Initially, a pre‐load of 0.2 N was applied to determine the initial depth. Subsequently, the maximum force of 200 N was applied, corresponding to a pressure of 40 MPa.

The adhesion strength of the electrode layers was also determined with the same ZwickiLine 2.5 kN machine (ZwickRoell), but fitted with a Z‐direction tensile strength setup. The procedure used for this test is similar to that described in the relevant literature [36]. Briefly, the electrode was placed between two planar, parallel sample holders; each sample holder was fitted with double‐sided adhesive tape (TESA Tesafix 5696 extra strong). Subsequently, a uniaxial compression stress of 2000 N was applied to the sample (with an area of 6.45 cm^2^) for 30 s. Afterward, the holders were pulled apart at a constant velocity. The adhesion strength was calculated based on the maximum tensile force F_max_ (N) and the sample area A (mm^2^) (see Equation (1)). The obtained adhesion strengths σ (N mm^−2^) were averaged from five measurements, and standard deviations were calculated accordingly.

(1) $$\sigma =\frac{F_{max}}{A}\;$$

### 2.4 Electrochemical Characterization in Symmetric EDLCs

The electrochemical characterization was performed using symmetrical coin cells (2032 standard coin cell type). First, the prepared electrode layers were punched into 12 mm discs (with an area of 1.131 cm^2^) by using a high‐precision Nogami hand‐held electrode puncher. Then, the electrodes were dried at 120°C under vacuum overnight (Buchi, pressure < 1 mbar) and transferred to an argon‐filled glovebox (LabMaster, Mbraun GmbH), with H_2_O and O_2_ content below 0.1 ppm. Then, the cells were assembled with a 1 mm thick stainless‐steel spacer, a stainless‐steel spring, and two paper separators were placed between the symmetric electrodes. The average active material loading of each punched electrode was approximately 6 mg cm^−2^. As an electrolyte, approximately 100 µL of 1 m Pyr_11_BF_4_ in ACN was used in each cell.

Cyclic voltammetry (CV) and electrochemical impedance spectroscopy (EIS) measurements were performed on a VMP‐3 multichannel potentiostat (BioLogic). For CV, scan rates of 1, 5, 50, 100, and 200 mV s^−1^ were applied within a cell voltage window of 0 to 3 V. The specific capacitance (C_s_) from CV plots was calculated using Equation (2):

(2) $$C_{s}=\frac{\int I\;dV}{m\;\Delta V\;s}\;$$

where ∫IdV is the integrated area under the CV plot, *m* is the mass of active materials, ΔV is the voltage window, and *s* is the scan rate.

EIS measurements were conducted in a frequency range of 1 MHz to 10 mHz with a voltage amplitude of 10 mV. Galvanostatic charge‐discharge (GCD) cycling tests and floating voltage tests were carried out using a Maccor Battery Tester 4300. All tests were conducted under controlled temperature of at 20 ± 2°C.

The GCD tests were started at a current density of 0.1 A g^−1^ based on the weight of active materials on both electrodes, and the current density was increased every 100 cycles up to 10 A g^−1^. Floating voltage tests were carried out at an upper voltage of 3 V at intervals of 25 h for a total of 500 h. After each voltage hold interval, five galvanostatic cycles were conducted to monitor capacitance and equivalent series resistance with a constant current of 0.1 A g^−1^. The specific capacitance of electrodes was calculated during the charge‐discharge steps via the following equation:

(3) $$C_{s}=\frac{I\;\Delta t}{m\;\Delta V}\;$$

where *I* is the discharge current, Δt is the discharge time, *m* is the mass of active material on both electrodes, and ΔV is the voltage window during discharge, excluding the voltage drop caused by internal resistance (IR) at the start of the discharge.

The equivalent series resistance (ESR) was determined form the voltage profiles by using Equation (4):

(4) $$\mathrm{ESR}\;=\frac{\;\Delta V}{I}\;$$

where *ΔV* is the IR drop at the beginning of the discharge curve, and I is the applied discharge current.

## 3 Result and Discussion

### 3.1 Effect of Cross‐linking on Binder Network

**FIGURE 1:**
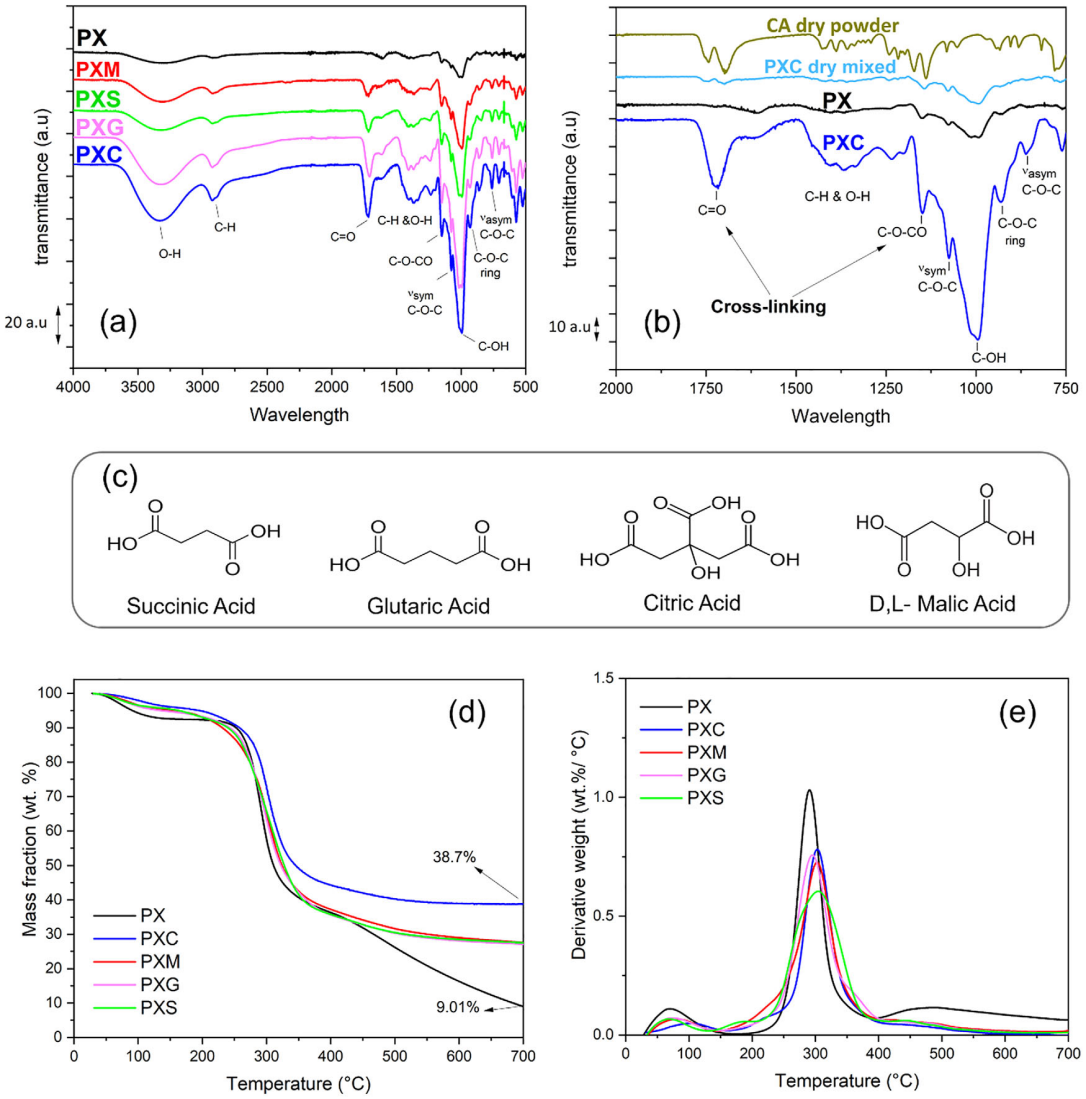
(a) Complete FTIR spectral profiles of PX, PXC, PXM, PXG, and PXS (b) Representative FTIR spectra (750–2000 cm^−1^) of PX and PXC polymer films compared with dry citric acid (CA) powder and mixed PXC, highlighting differences between PX and PXC polymer films and dry powder of CA and mixed PXC (c) Chemical structure of cross‐linking agents (d) TGA thermograms of the prepared polymer binder films (PX, PXC, PXM, PXG, and PXS) showing residual mass as a function of temperature (e) Corresponding mass loss derivatives of the TGA curves as a function of temperature.

Cross‐linking via esterification with carboxylic acid has been largely reported in literature [14, 24, 25, 26, 27, 28, 29, 30]. However, in most works the thermally activated condensation reaction is performed at relatively high temperature, up to 150°C [25, 26, 27]. This is not only energy demanding, but also potentially incompatible with industrial manufacturing lines, whose heating units typically operate at a maximum 120°C. It is therefore important to verify whether binder cross‐linking can take place at a lower temperature, e.g., 80°C, as chosen in this work. In order to determine the occurrence of the esterification reaction, FTIR analysis was conducted on the prepared polymer binders and cross‐linking agents. The FTIR spectra collected in the 4000 – 400 cm^−1^ range for all prepared samples (after baseline correction and vertical shifting) are presented in Figure 1a. The spectra of PX, PXC, CA powder, and a dry mixed PXC blend (PS: XG(3:1) + CA(10 wt.%) non‐crosslinked) with the same composition of PXC are also included in Figure 1b for better comparison. The FTIR spectra of pure PS and XG powders are provided in the Figure S1 for completeness. The FTIR spectra of the PX sample exhibit characteristic peaks of polysaccharide polymers, such as the broad O‐H stretching band between 3100 and 3600 cm^−1^, the C─H peak around 2924 cm^−1^, C═O (1617 cm^−1^), and C─O (995 cm^−1^) [24, 26, 37]. The spectrum of CA features the typical peaks of C═O stretching in carboxylic acids at 1742 cm^−1^ and 1700 cm^−1^ [25, 38]. The non‐crosslinked dry mixed PXC blend displays peaks similar to those of the PX spectra, with additional features at 1701 cm^−1^ and 1750 cm^−1^ that can be assigned to the C═O group in citric acid. However, these additional peaks show lower intensity, which could be expected due to the lower weight fraction of citric acid in this dry mixture. More interestingly, the FTIR spectra of cross‐linked PXC display significant changes with the emergence of a new peak at 1728 cm^−1^, while the peaks at 1701 cm^−1^ and 1750 cm^−1^ disappear (Figure 1b). Additionally, the peak between 1150 and 1250 cm^−1^ associated with C─O─CO asymmetric stretching in ester groups becomes substantially more pronounced. Similar features are observed in the spectra of other cross‐linked polymers such as PXM, PXG, and PXS (see Figure 1a) [24]. However, the intensity of the cross‐linking fingerprint peak at 1728 cm^−1^ is more pronounced in PXC spectra compared to other binders, likely due to the presence of more available sites for esterification reactions in citric acid, as shown by the chemical structure of cross‐linking agents in Figure 1c. These are clear evidence that the cross‐linking has taken place, as also observed in prior studies [14, 27, 39, 40]. Therefore, it is demonstrated that it is not necessary to excessively heat the sample to 150°C. A full temperature optimization study was not carried out in this work, as it would require a systematic evaluation across several temperatures, drying durations, and mechanical/electrochemical outcomes. Such optimization is going to be the subject of future work. Here, 80°C is identified as a practical and effective processing temperature that enables measurable esterification while remaining compatible with industrial electrode drying setups. It should be noted that esterification is a dehydration type equilibrium reaction, so the water content in the electrode directly influences the cross‐linking. In electrode preparation, the drying step at 80°C removes most of the water in the slurry, and FTIR already indicates that part of the ester network forms at this stage. After calendaring and punching, the electrodes undergo the routine high‐vacuum drying at 120°C (< 1 mbar, overnight) before cell assembly. This step removes the remaining bound water, and the electrodes are transferred directly into the Ar glovebox (H_2_O and O_2_< 0.1 ppm), which provides the very dry conditions required for EDLC assembly. Under these conditions, the equilibrium shifts further toward ester bond formation.

TGA was also performed to investigate the impact of cross‐linking agents on the thermal stability of the binder formulations. Figure 1d,e presents the TGA thermograms of the polymer blends with and without cross‐linking agents. For completeness, the TGA results of the polymer binders are summarized in Table S2, while those of the pure components (PS, XG, and CA) are provided in Figure S2.

All prepared polymer blends show an initial mass loss up to ca. 150°C, likely attributed to the removal of residual water [24, 25]. The initial mass loss of the non‐cross‐linked polymer (PX sample) is approx. 7.4%, while cross‐linked polymers exhibit mass loss of around 3–5% (3.8% and 4.5% PXC and PXM, respectively). After heating to 700°C, the cross‐linked samples (PXC, PXM, PXG, and PXS) display about 20–30% lower weight loss than the non‐cross‐linked PX sample. Moreover, Figure 1e illustrates that after water evaporation, the thermal degradation of the non‐cross‐linked PX binder occurs at approx. 290°C (T_d_, Table S2), while the degradation of cross‐linked samples occurs at slightly higher temperature. For instance, PXC shows a T_d_ = 303°C (+13°C compared to PX) and PXM a T_d_ = 302°C (+12°C compared to PX). Overall, TGA results evidence that cross‐linking improves thermal resistance of the polymer binder blend.

### 3.2 Cross‐linked EDLC Electrodes: Morphology and Mechanical Properties

**FIGURE 2:**
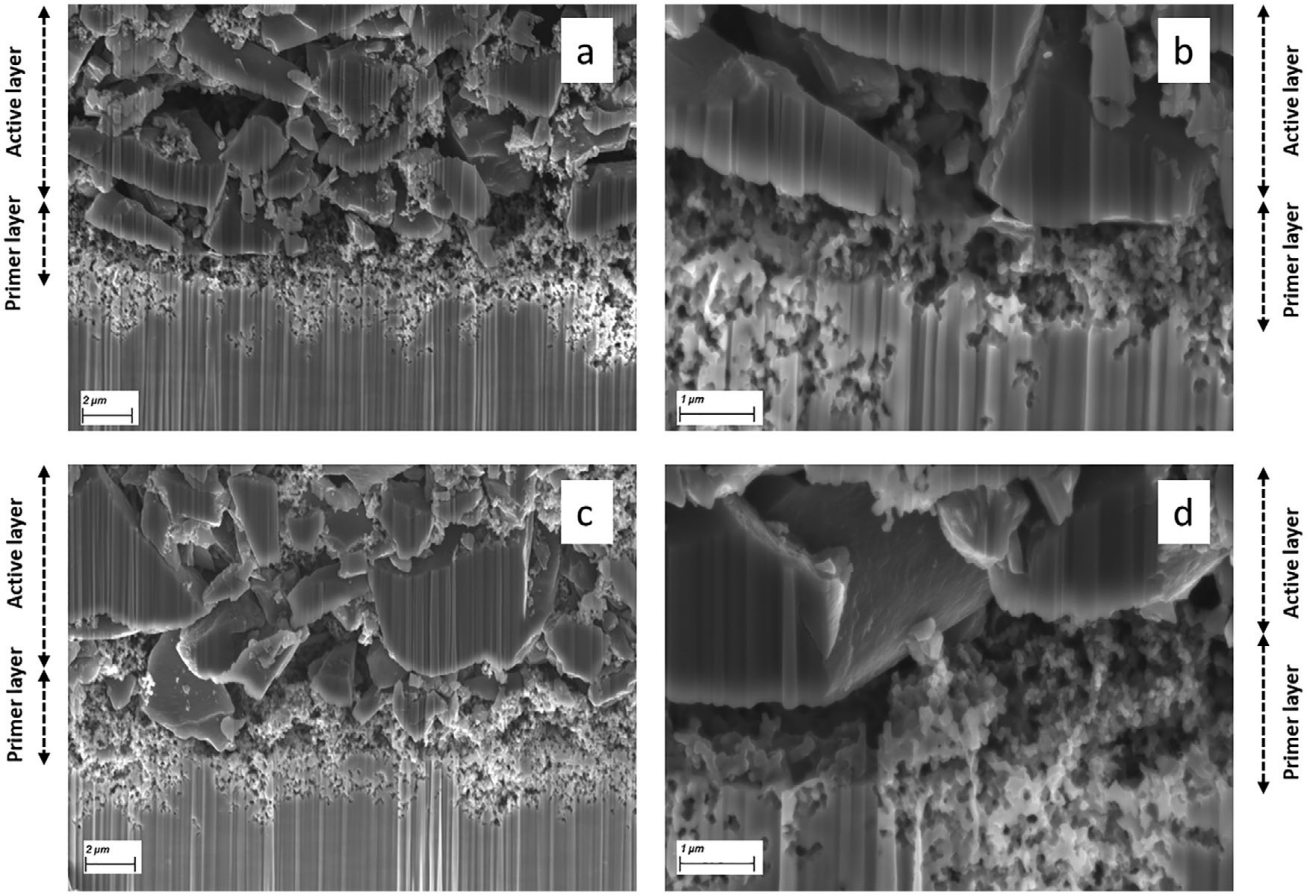
The SEM micrographs of electrodes with crosslinked (PXC) and non‐crosslinked binder (PX) at two magnification levels. (a, b) Electrode with PX binder;(c, d) electrode with PXC binder.

SEM micrographs were collected to investigate the morphological difference between cross‐linked and non‐cross‐linked electrodes. Surface morphology images of electrodes prepared with different cross‐linking agents (CLAs) are provided in Figure S3. No evident difference can be noticed among the samples. Therefore, for the sake of brevity, PX and PXC are selected for a more detailed comparison of the cross‐section. The aim is to characterize the boundary between the primer and active layer and their connection to the current collector. FIB‐SEM cross‐section images of electrodes with PXC and PX binders are presented in Figure 2. The images highlight large, irregularly shaped particles of the active material (CG) and finer particles of carbon black (C45). Clearly distinguishing the binder is not possible. In both cases, the particle distribution appears uniform, as does the inter‐particle porosity. In addition, the boundary between the active and primer layer can be well recognized. The thickness of the primer layer (approximately 10 µm right after casting) is reduced to 2–3 µm, likely due to the calendaring of the electrodes, indicating a strong interpenetration of the two layers. This intimate contact can facilitate electron transport across the current collector/electrode interface, which is important for achieving high‐performance and low ESR. At higher magnification (Figure 2b,d), the interaction between primer and active layer becomes even more evident. However, the specific components on the surface of the CG particles cannot be reliably identified. In summary, no obvious morphological differences between cross‐linked (PXC) and non‐cross‐linked (PX) electrodes are visible, which is likely due to changes in the binder network and particle interactions at the nanoscale that are below the resolution of the SEM.

**FIGURE 3:**
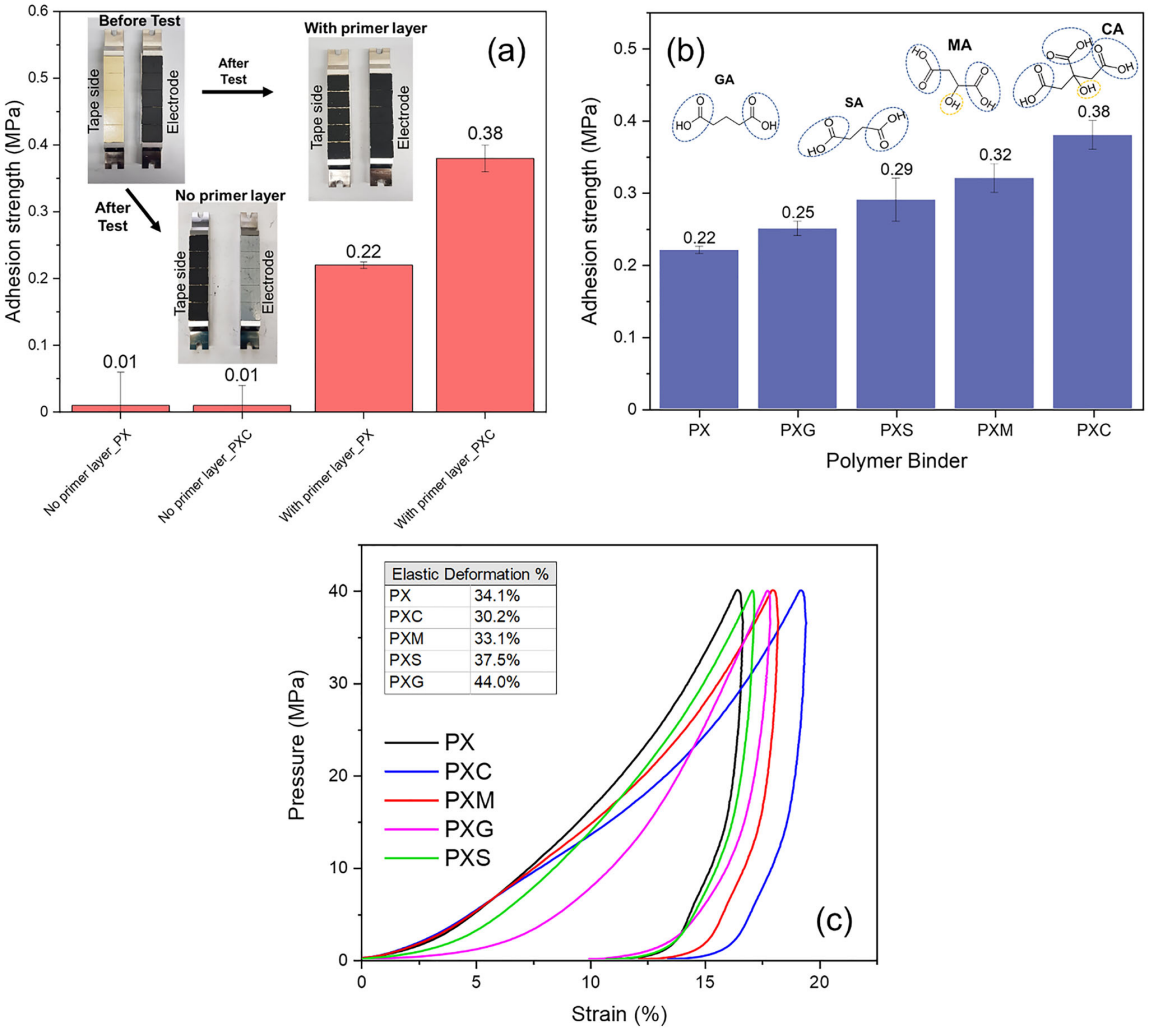
Calculated adhesion strength of electrodes (a) with two different binders, PX and PXC, with and without a primer layer; (b) with primer layer for PX, PXC, PXM, PXG, and PXS electrodes. (c) compression test results of PX, PXC, PXM, PXG, and PXS electrodes with calculated relative elastic deformation.

To better quantify the impact of primer layer and cross‐linking on the mechanical properties of electrodes, a comparative study was conducted. Four electrodes were prepared: two with PX binder and two with PXC binder. Within each binder group, one electrode included the primer layer, while the other did not. The adhesion strength of such electrodes was measured by the Z‐direction tape test, as shown in Figure 3a. As it can be observed from the plot, in the absence of a primer layer, the maximum tensile force required to peel off the electrode from the current collector is less than 10N (σ less than 0.015 MPa), indicating a very poor adhesion and no detectable influence of cross‐linking. Moreover, the results of the tests performed on electrodes without the primer layer were very scattered and always associated with a large standard deviation, which can be attributed to adhesion failure of the test caused by the poor interaction between the coating layer and the current collector, as mentioned by Haselrieder [36]. On the other hand, for the electrodes with the primer layer, the maximum tensile force required for peeling is substantially larger. More importantly, the influence of cross‐linking becomes very evident. The electrodes with PX binder and the primer layer show a maximum tensile force of 140.8 N (σ = 0.21 MPa), while those with the PXC binder and the primer layer display an even higher maximum tensile force of 222.8 N (σ = 0.35 MPa). This significant increase in tensile force demonstrates the synergic effect of both the primer layer and cross‐linking in enhancing the adhesion strength between the active layer and the current collector. Our findings align with the work of Diehm et al., who found the presence of a primer layer significantly improved the mechanical stability of electrode materials [34]. Similarly, Lee et al. reported that a thin adhesive polymer layer can enhance adhesion and cyclic performance of electrodes [35]. Representative images of the adhesion tape test setup and post‐test condition are provided in Figure 3a to visually support the mechanical data. For the electrode without a primer layer, the current collector is clearly visible after the adhesion tape test, indicating that the active layer detached completely from the current collector due to adhesion failure at the electrode‐current collector interface. In contrast, the electrode with a primer layer shows no visible current collector, suggesting that the detachment occurs partly within the electrode structure itself, yet with sufficient cohesion and in agreement with the improved adhesion strength data. In general, adhesion failure refers to delamination at the interface between the electrode coating and the current collector, while cohesion failure occurs within the electrode coating due to insufficient internal strength or binder distribution [36].

To understand the influence of different cross‐linking agents on the mechanical properties of the electrodes, the adhesion tape tests were extended to PXG, PXS, and PXM, too. Figure 3b summarizes the adhesion strength values of electrodes with the primer layer and different binder formulations (averaged from five measurements).

The plot shows a clear correlation between the adhesion strength and the presence of cross‐linking agents. For instance, the electrode with non‐cross‐linked PX binder demonstrates an adhesion strength of 0.22 MPa, while the cross‐linked ones display 0.25, 0.29, 0.32, and 0.38 MPa for PXG, PXS, PXM, and PXC, respectively. Interestingly, there seems to be a correlation between the number of ‐OH available for esterification and the adhesion strength. In fact, CA (3x ‐OH) provides more adhesion than SA, MA, and GA (2x ‐OH). The results clearly demonstrate that cross‐linking significantly enhances electrode adhesion, with CA creating the most effective binder network. This can be expected to improve the electrochemical performance in the long term.

To obtain a more comprehensive characterization of the mechanical properties, compression tests were performed as well. The corresponding elastic deformation of electrodes with different binders is shown in Figure 3c. Cross‐linked electrodes consistently required a lower indentation force at all depths compared to the PX electrode in order to reach the same penetration depth. Interestingly, elastic deformation values vary as a function of the CLA and may be correlated to their different chemical structure. For example, PXC and PXM electrodes show lower elastic deformation values (30.2% and 33.1%, respectively) compared to PX. This is attributed to increased binder network density due to the higher cross‐linking potential of CA and MA. However, PXS and PXG display higher elastic deformation values (37.5% and 44%, respectively), which can be related to the longer alkyl chain separating the two carboxylic acid units of SA and GA, thus creating more flexible structures. These results align well with the molecular characteristics of the cross‐linking agents: CA, a tri‐carboxylic acid, offers more cross‐linking sites due to its three carboxyl groups and an additional hydroxyl (OH) group, which creates a denser and stiffer binder network. Furthermore, its OH group contributes to enhanced network stability through intermolecular hydrogen bonding interactions with the polysaccharide chains. MA offers moderate cross‐linking potential, as it contains only two carboxyl groups and one hydroxyl group, allowing it to enhance the strength of the binder network through the same dual mechanisms of covalent bonding and non‐covalent hydrogen bonding interactions. This combined effect explains the superior mechanical performance of PXM and PXC compared to other dicarboxylic acids. In fact, GA and SA are dicarboxylic acids with only two reactive carboxylic units and no hydroxylic group, which limits their cross‐linking ability to two covalent ester bonds and lacks the effect of OH hydrogen bonding. GA creates a more flexible structure with a longer five‐carbon chain. Also, PXS displays an intermediate flexibility resulting from a compromise between its cross‐linking density and the shorter chain length of SA. These results highlight how the electrode's mechanical properties can be tuned by adjusting the cross‐linking structure of the binders.

### 3.3 Electrochemical Performance in Symmetric EDLC

**FIGURE 4:**
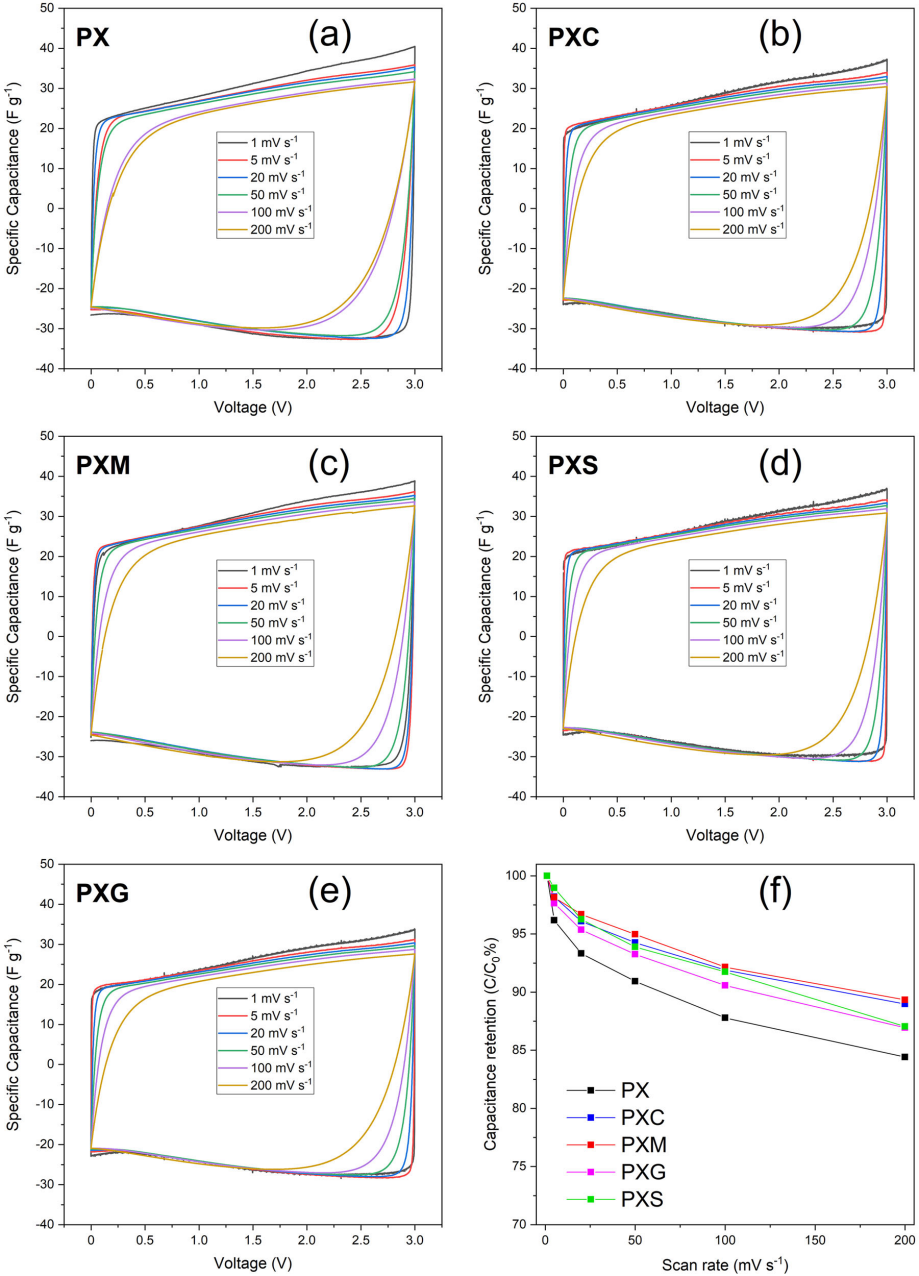
CV plots of symmetric EDLCs with (a)PX; (b) PXC; (c) PXM; (d) PXS; (e) PXG binder materials, and (f) related capacitance retention as a function of scan rate.

The electrochemical response of electrodes with different binders was first evaluated in symmetric EDLCs by CV in the potential window of 0 to 3 V and scan rates ranging from 1 to 200 mV s^−1^, as presented in Figure 4. The voltammograms for all EDLCs display an almost ideal rectangular shape typical of double‐layer capacitive behavior (Figure 4a–e). The absence of any faradaic (redox) feature demonstrates that the binders, especially the cross‐linked ones, do not cause any parasitic side‐reaction that may harm the electrochemical reversibility. In all cases, the EDLCs deliver specific capacitance values of around ​ 28 to 30 F g^−1^ at 1 mV s^−1^. The slight differences are likely attributed only to small deviations in electrode mass. Figure 4f shows the capacitance retention of cells with different binders at increasing scan rate. Clearly, all cells with cross‐linked binders exhibit capacitance retention greater than 86% and up to 90% at 200 mV s^−1^, while the cell with PX electrodes (non‐cross‐linked polymer binder) shows the lowest retention of “only” 84%. To exclude any influence from the binder or the primer layer on the electrochemical response, additional control measurements were performed. First, CV measurements were performed on EDLC containing electrodes with the primer layer only (PX binder, Figure S4). It is evident that the primer layer itself does not affect the overall capacitance (areal capacitance of 0.75 mF cm^−2^). Second, the interaction between the binder and the electrolyte was evaluated by coating solutions of PX and PXC binders directly onto the etched Al current collector and testing them as binder‐only electrodes. The CV curves remained smooth and stable, with no additional peaks or any indication of binder degradation. This confirms that both the binder and the primer are electrochemically inert. The complete CV data and measurement parameters are provided in Figure S5.

**FIGURE 5:**
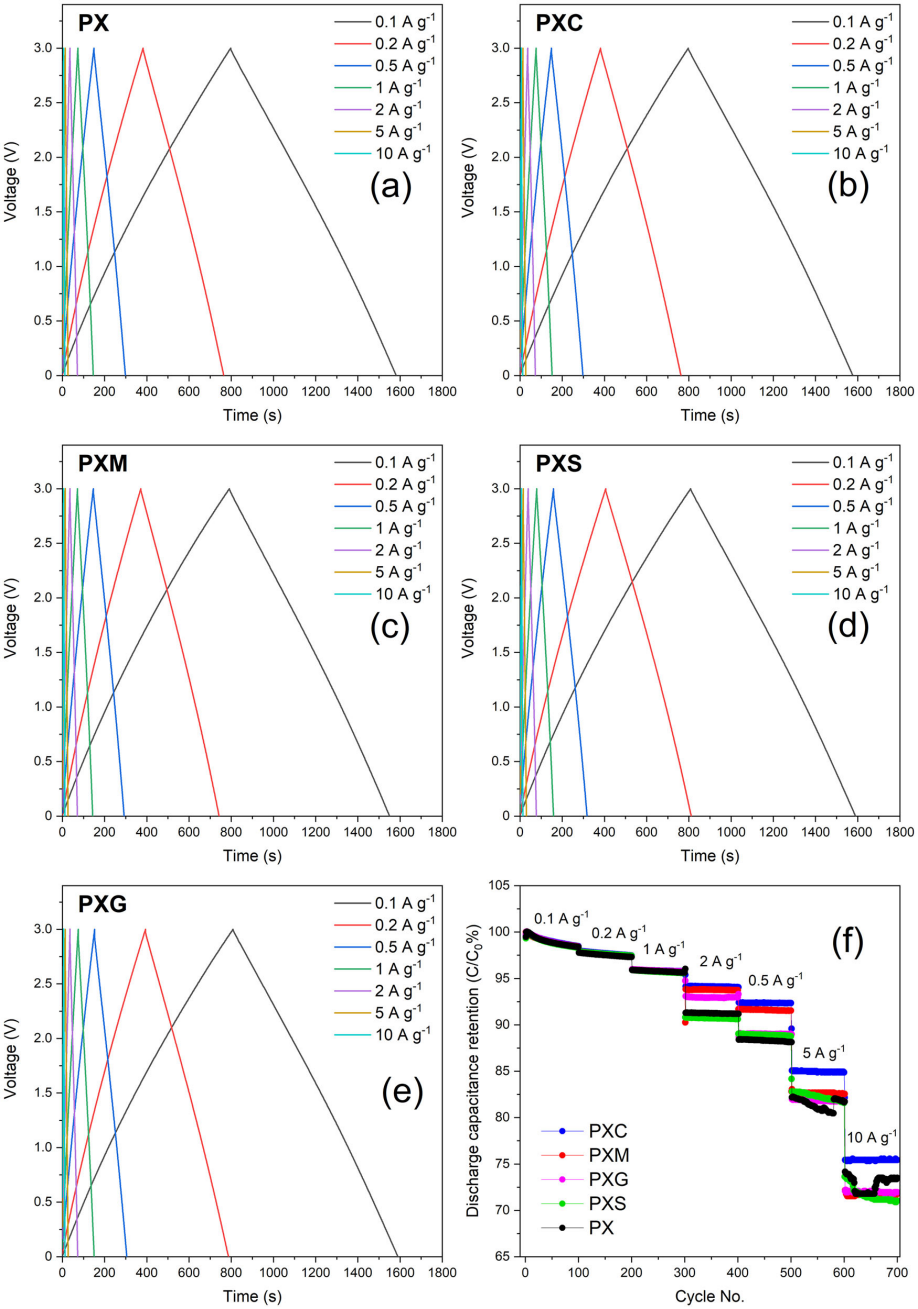
Voltage profiles of EDLC cells with (a) PX; (b) PXC; (c) PXM; (d) PXS; (e) PXG at various current densities in ranging from 0.1 to 10 A g^−1^, and (f) corresponding capacitance retention.

Afterward, the EDLCs were subject to GCD tests within the same voltage window of 0–3 V and at current densities ranging from 0.1 to 10 A g^−1^ (based on the weight of active materials on both electrodes). As shown in Figure 5a–e, all cells display voltage profiles with the typical triangular shape expected when charging/discharging an ideal capacitor. The linear charging and discharging behavior of the GCD plots is also observed under a high current density of 10 A.g^−1^, confirming efficient electronic and ionic transport within the electrodes. This highlights the low resistance and good ionic accessibility of these electrodes fabricated with polysaccharide binders. The capacitance retention of the different cells at different specific currents (calculated by using Equation (3)) is shown in Figure 5f. Of course, the capacitance decreases with increasing current density in a similar manner as previously in CV tests at different scan rates. Most remarkably, the cross‐linked electrodes show improved retention across all current densities. Even at high current density (10 A g^−1^), a capacitance retention of over 70% is observed. To further verify the durability of the binder and electrode, the cycling performance was evaluated over 1000 cycles. The results are provided in Figure S6 and confirm the stability of the electrodes.

**FIGURE 6:**
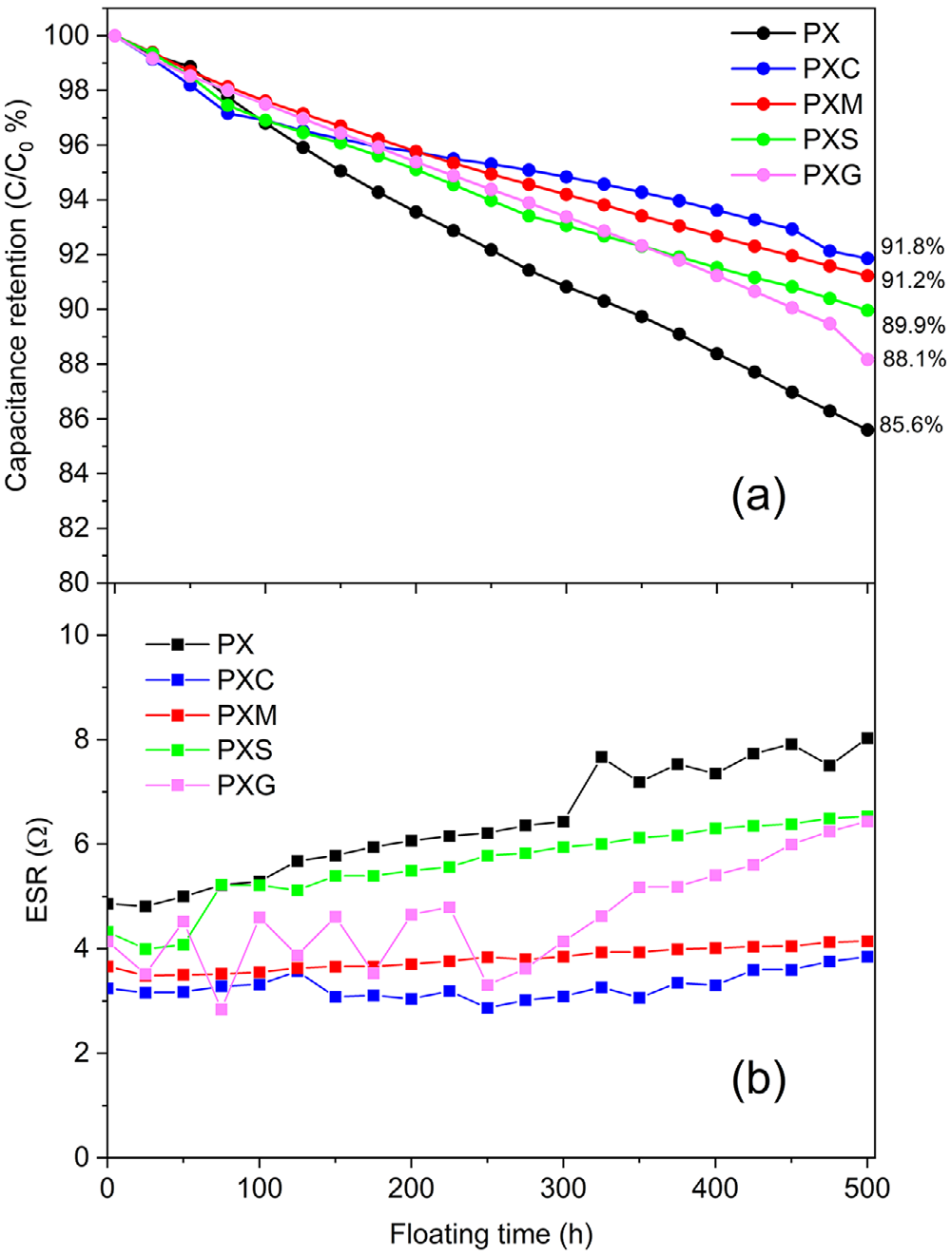
(a) Specific capacitance retention and (b) ESR evolution of EDLCs based on PX, PXC, PXM, PXS, and PXG electrodes as a function of floating time.

Floating voltage tests (also known as voltage hold) were also conducted to investigate the influence of cross‐linking on long‐term ageing at high voltage. The specific capacitance and ESR were measured every 25 h during a 500 h test at a constant maximum cell voltage of 3 V (by using Equations (3) and (4)) as reported in Figure 6.

The results show that the EDCL with non‐cross‐linked PX electrodes retains 84.3% of its initial capacitance after 500 h. On the other hand, those including cross‐linked electrodes display substantially higher retention. In fact, PXC and PXM enable the highest capacitance retention of more than 91%, while PXG and PXS allow slightly lower retention levels of 88.1% and 89.9%, respectively. Most interestingly, the high voltage stability appears to be directly correlated with the adhesion strength of the employed electrodes. Indeed, float voltage stability and adhesion strength follow the same sequence PXC>PXM>PXS>PXG>PX. As shown in Figure 6b, the ESR of the fresh cells (t = 0) follows pretty much the same pattern (but of course, inverted: PX>PXS≈PXG>PXM>PXC). Furthermore, the plot displays a relatively linear and stable behavior for PXC‐ and PXM‐based EDCLs during the long‐term stability test. Differently, a more pronounced ESR increase is observed for the cells featuring electrodes with lower adhesion strength (i.e., PXG, PXS, and in particular, PX).

The floating voltage tests clearly highlight a pronounced difference in stability between cross‐linked (PXC, PXM, PXS, and PXG) and non‐cross‐linked electrodes (PX). These results indicate the role of cross‐linking agents in enhancing the high voltage stability of EDLCs, which can likely be attributed to improved mechanical strength of the electrodes demonstrated by the adhesion tape test results.

**FIGURE 7:**
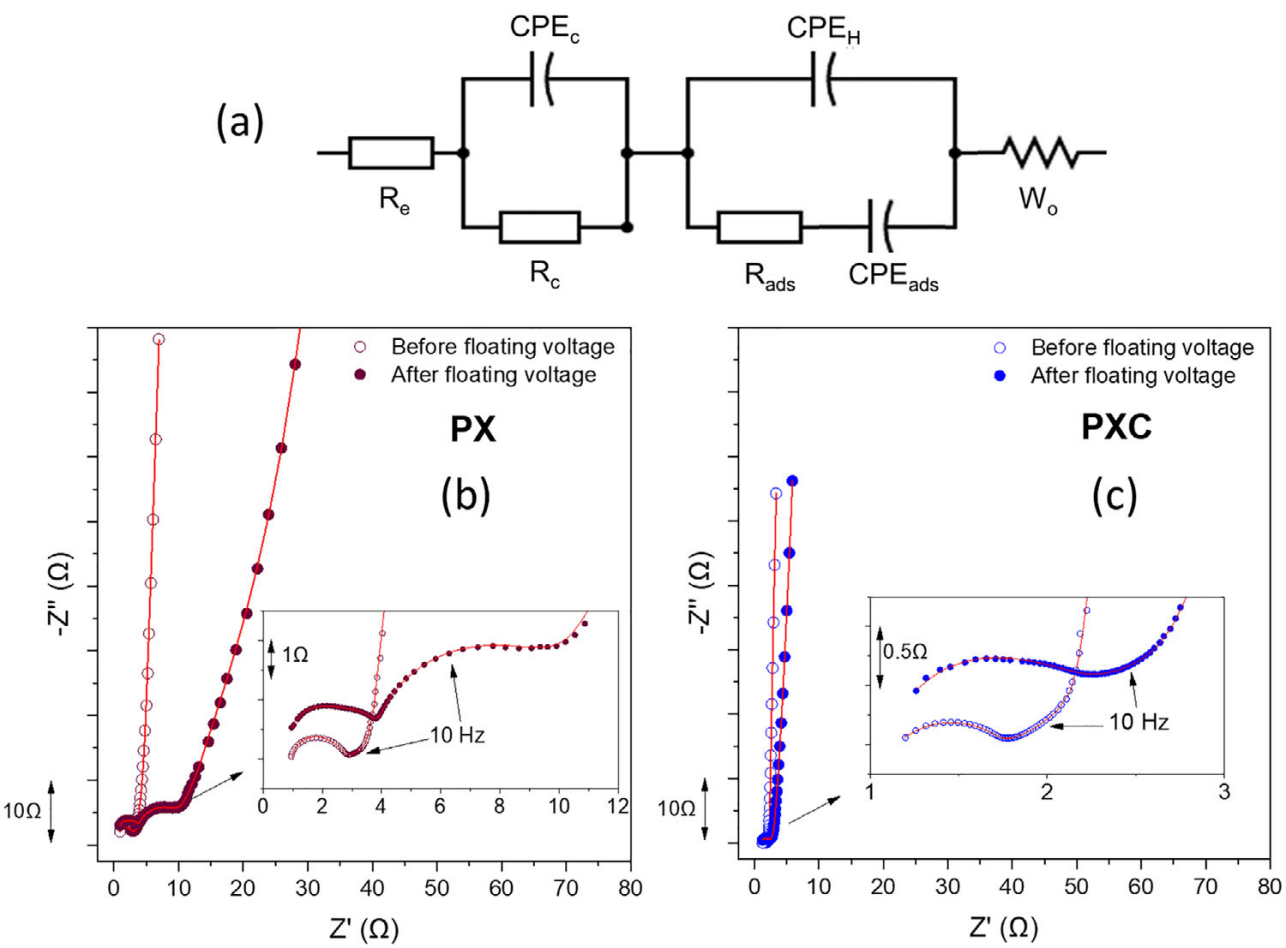
(a) Equivalent circuit model used to fit the EIS spectra. (b–c) Nyquist impedance plots of EDLC cells with PX and PXC binders, recorded before and after the floating voltage test.

To further understand the role of binder cross‐linking, electrochemical impedance spectroscopy (EIS) was carried out on EDLC cells with PX‐ and PXC‐based electrodes. The EIS spectra were collected in the frequency range of 1 MHz to 10 mHz, both before (fresh) and after a 500 h floating voltage test. The results are plotted in the form of Nyquist plots in Figure 7, along with the equivalent electric circuit models used to fit the experimental spectra (fits shown by red lines). The most important data resulting from EIS analysis are also listed in Table 1. For completeness, EIS spectra and fitted parameters for cells using PXM, PXG, and PXS binders are provided in Figure S7 and Table S2.

**TABLE 1:** The main result of Nyquist plots of binders after fitting with the equivalent circuit model.

Sample	R_e_(Ω)	R_c_(Ω)	R_ads_(Ω)
PX before floating voltage	0.84 ± 0.01	1.33 ± 0.02	1.48 ± 0.01
PX after floating voltage	0.88 ± 0.01	3.03 ± 0.01	4.84 ± 0.03
PXC before floating voltage	1.18 ± 0.02	0.75 ± 0.01	0.57 ± 0.01
PXC after floating voltage	1.28 ± 0.01	1.02 ± 0.01	0.76 ± 0.01

The EIS response of EDLCs typically displays three characteristic behaviors at high, middle, and low frequencies. In the high frequency range, a semi‐circle, which can be assigned to the contact resistance at the interface of the electrode materials and current collectors, is typically observed [41, 42]. The diameter and shape of the semicircle are related to electrode porosity and the electrolyte resistance in the pores of the electrodes. In the mid‐frequency region, a line with a slope of 45° is often visible, which is known as Warburg impedance (W_o_), and is related to ion diffusion and limitation of ion transport in the porous electrode. At low frequency, the plots show a near vertical line related to the capacitive behavior of the electrodes [43, 44]. Since active material and electrolyte compositionares the same for all tested cells, any difference in internal resistance can be mainly related to the binder composition and the influence of cross‐linking agents.

Figure 7a displays the equivalent circuit model used to fit the spectra of EDLC cells with PX and PXC electrodes. Due to the porous nature of the electrodes and their heterogeneous surface, the double‐layer does not behave as an ideal capacitor. Instead, it is modeled using a constant phase element (CPE), which accounts for non‐ideal behavior. The proposed equivalent circuit consists of three main elements. First, R_e_ represents the bulk resistance of the electrolyte and is identified by the intercept of the high‐frequency semicircle with the real axis(Z). Second, the electrode contact resistance (R_c_), which accounts for the intrinsic resistance of the electrode, including the contact resistance between the active layer and the current collector, is modeled in parallel with CPE_c_, representing the non‐ideal capacitive behavior of the porous electrode matrix. The third section of the model captures interfacial processes. In the absence of faradaic processes, as is the case in EDLCs, no true charge‐transfer resistance is expected. However, interfacial impedance may still arise due to sluggish ion dynamics, pore accessibility, or incomplete double‐layer formation under certain conditions. This includes the Helmholtz capacitance (C_H_), representing the compact layer formed by solvated ions electrostatically attracted to the electrode surface (outer Helmholtz plane), and the adsorption capacitance (C_ads_) associated with specifically adsorbed ions. These capacitive components are modeled in parallel with a resistance, which is called adsorption resistance (R_ads_) and reflects the impedance linked to ion rearrangement or specific adsorption kinetics at the interface. The last component is W_o,_ which is the Warburg impedance [43, 45].

The fresh cell with PX binder cell exhibited a R_e_ of 0.82 Ω, a contact resistance (R_c_) of 1.33 Ω, and an adsorption resistance (R_ads_) of 1.48 Ω. In contrast, the cell with cross‐linked PXC binder electrodes had a slightly higher initial R_e_ of 1.18 Ω, but notably much lower R_c_ (0.55 Ω) and R_ads_ (0.57 Ω). The PXC binder greatly reduces the interfacial and contact resistances. This indicates that the cross‐linked PXC binder improves electrical connectivity and minimizes ion transport resistance within the electrode structure. The smaller semicircle in the Nyquist plot of the PXC cell supports this conclusion, implying stronger adhesion and cohesion within the electrode composite. Dsoke et al. also reported that the size of this semi‐circle can be reduced with optimizing the electrode fabrication process [41]. The much lower R_ads_ for the PXC based cell indicate minimized resistance to ion penetration within the porous carbon network. In other words, the cross‐linked binder can cause minimal pore blockage, allowing efficient ion diffusion into micropores. Similarly, Jeżowski et al. found that using a starch‐based binder significantly lowered the equivalent distributed resistance (EDR) of EDLC electrodes, which was attributed to noticeably lower pore clogging by this type of binder [22].

After the floating voltage test, both types of EDLC cells exhibited some increase in impedance, but the extent of degradation differed dramatically between the two binder systems. In the PX‐based cell, the EIS spectrum showed a combination of two semicircles in the high and mid‐frequency regions. Notably, part of the resistance that was initially in the high‐frequency range shifts toward the mid‐frequency range (10 Hz to 1 kHz), indicating changes in the interfacial properties and possibly the emergence of additional resistive elements such as degraded contact points or resistive surface films. Specifically, the PX‐based cell exhibited a slight increase in R_e_ to 0.88 Ω (+0.06 Ω), whereas R_c_ and R_ads_ increased significantly to 3.03 and 4.84 Ω, respectively, more than double its initial value. In contrast, the PXC binder cell displayed much more stable impedance. R_e_ increased to 1.28 Ω (+0.10 Ω), R_c_ to 1.02 Ω (the initial 0.75 Ω), and R_ads_ increased slightly to 0.76 Ω (33% rise from 0.57 Ω). Even after the long‐term floating voltage test, the PXC‐based cell's resistances remained far lower than those of the PX‐based one.

In the fresh state, only one semicircle is visible because the R_C_ and R_ads_/C_ads_ processes have very similar relaxation times and overlap in the high‐frequency region. Both resistances are small (e.g., for PXC: R_C_ = 0.75 Ω and R_ads_ = 0.57 Ω), so the two processes merge into a single depressed semicircle even though both are present. After the floating voltage test, surface changes and mild aging affect these components differently and increase their separation. For example, in the PX electrode, R_C_ increases to 3.03 Ω and R_ads_ to 4.84 Ω, resulting in two distinct relaxation times and the appearance of a second semicircle. This behavior is consistent with EDLC literature [45], where overlapping interfacial processes appear as one semicircle when the resistances are small. The resistance increase during the EDLC floating voltage test can be linked to changes in the electrode/binder matrix, leading to poorer conductivity, or contact problems caused by reduced electrode integrity. Possible failure modes include binder decomposition (due to electrochemical oxidation or reactions with electrolyte) and loss of adhesion and cohesion. Any of these would lead to loss of contact between carbon particles and the current collector, explaining the large R_c_ increase of the PX cell (from 1.33 to 3.03 Ω). These large increases point to serious deterioration of the electrode microstructure and binder effectiveness in the electrode. For instance, if the binder can no longer hold the active material tightly, micro‐cracks or delamination can occur, dramatically increasing interfacial resistance. Also, the increase of R_ads_ in the PX electrode can suggest that ion movement into pores became severely blocked after the test. This could be due to pore blockage by detached binder fragments or precipitated decomposition products of the electrolyte that make it harder for ions to access the active material surface.

## 4 Conclusion

This work investigated a sustainable binder system made from a PS and XG blend, cross‐linked using different carboxylic acids (citric, malic, succinic, and glutaric), and showed their strong influence on both the mechanical and electrochemical performance of EDLC electrodes. FTIR and TGA confirmed the occurrence of cross‐linking via esterification at a relatively low temperature (80°C), which is compatible with common industrial setups and sufficient to drive the esterification reaction to a degree that yields improved mechanical properties of the electrodes. A full optimization of the cross‐linking temperature remains an interesting direction for future investigation. The comparison between various cross‐linking agents shows a clear structure‐property relationship that directly plays an important role in electrode performance. In particular, CA, with three carboxyl groups and a hydroxyl group, forms the densest network and leads to the highest adhesion strength and lowest elastic deformation. MA provides a similar effect that supports both esterification and hydrogen bonding. In contrast, SA and GA, with two reactive groups and longer chains, more flexible alkyl chains, form less rigid networks that result in weaker adhesion but higher elasticity. These molecular structural differences explain the different behaviors observed in mechanical characterizations, where PXC delivered the highest strength, while PXS and PXG with longer alkyl chains allowed more elastic deformation.

The same trend was observed in electrochemical performance. Electrodes with stronger adhesion (PXC, PXM) showed lower interfacial resistance, superior rate capability, and higher capacitance retention during long‐term floating voltage tests. On the other hand, the more flexible but weaker cross‐linked binders (PXS, PXG) provide only moderate improvements over the non‐cross‐linked reference (PX) and cannot deliver the same long‐term stability.

Overall, the correlation between adhesion strength, the chemical structure of cross‐linking agents, and electrochemical performance highlights that the right choice of cross‐linking agent is key to designing sustainable, water‐processable binders for EDLCs that combine both mechanical integrity and stable long‐term operation in EDLCs.

## Conflicts of Interest

The authors declare no conflicts of interest.

## Supporting information




**Supporting File**: advs73510‐sup‐0001‐SuppMat.docx.

## Data Availability

The data that support the findings of this study are available from the corresponding author upon reasonable request.;
