# Supplementary material for: Enhancing Mechanical and Electrochemical Stability of EDLC Electrodes via Crosslinked Polysaccharide Binder Blends
Source: Adv Sci (Weinh). 2025 Dec 23;13(13):e20621. doi: 10.1002/advs.202520621 (PMC12955855; doi:10.1002/advs.202520621)
Supplement: Supplementary file 1 — Supporting File: advs73510‐sup‐0001‐SuppMat.docx. [file ADVS-13-e20621-s001.docx]

**Supporting information**

# Enhancing Mechanical and Electrochemical Stability of EDLC Electrodes via Crosslinked Polysaccharide Binder Blends

Mahdi Karimi Jafari^1,2^, Rupesh Singh^1,2^, Stefano Passerini^3^, Alberto Varzi^1,2,*^

^1^ Karlsruhe Institute of Technology (KIT), P.O. Box 3640, 76021 Karlsruhe, Germany

^2^ Helmholtz Institute Ulm (HIU), Helmholtzstrasse 11, 89081, Ulm, Germany

^3^ Austrian Institute of Technology (AIT), Center of Transport Technologies, Giefinggasse 2, 1220 Vienna, Austria

*corresponding author: [alberto.varzi@kit.edu](mailto:alberto.varzi@kit.edu)

Table S 1- List of prepared polymer binders blend with nominal weight composition.

| **Sample name** | **PS (wt.%)** | **XG (wt.%)** | **Cross-linking agent (wt.%)** |
| --- | --- | --- | --- |
| PX | 75 | 25 | - |
| PXC | 67.50 | 22.50 | 10 (CA) |
| PXG | 67.50 | 22.50 | 10 (GA) |
| PXM | 67.50 | 22.50 | 10 (MA) |
| PXS | 67.50 | 22.50 | 10 (SA) |


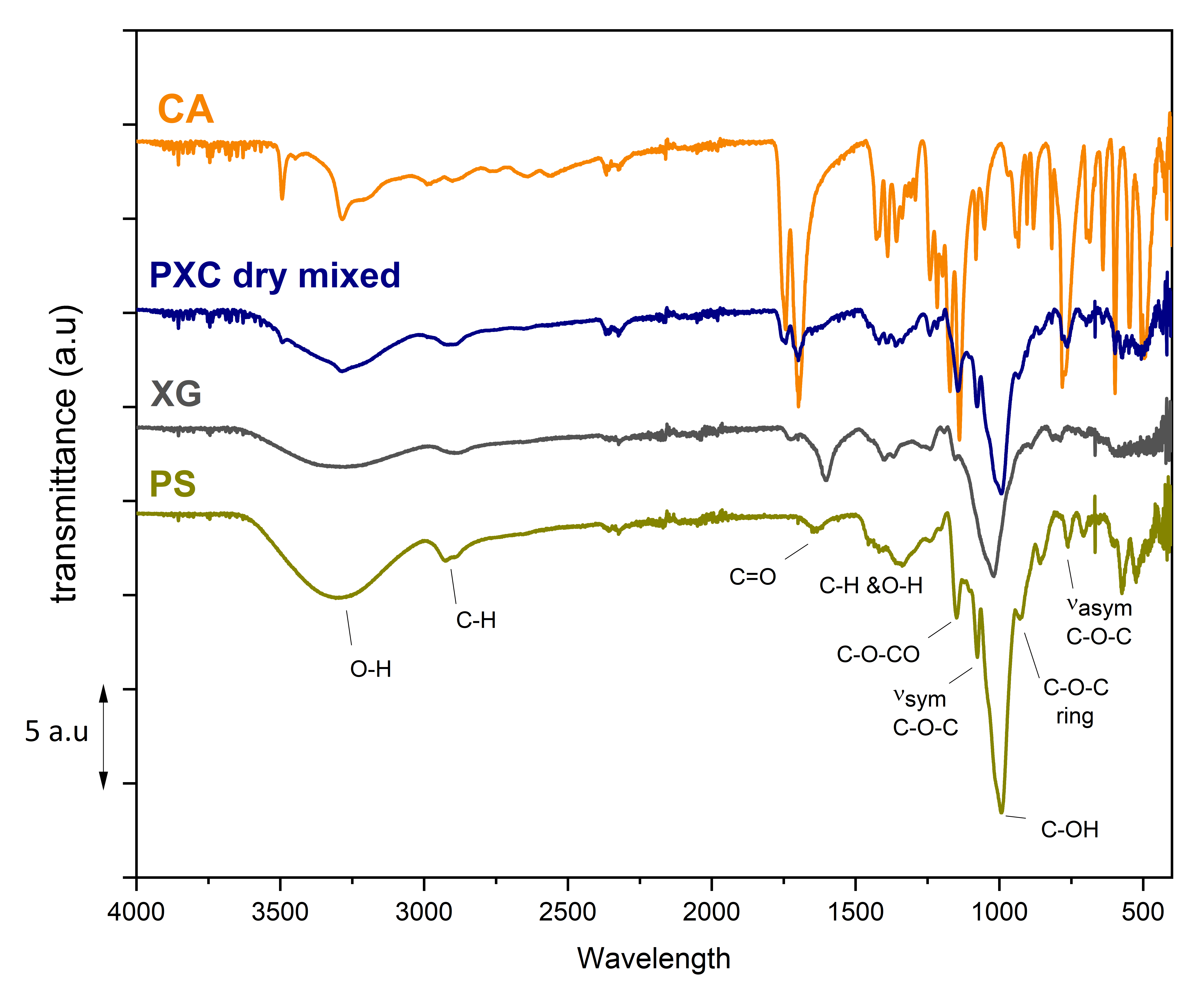

Figure S 1- FTIR spectra of PS, XG, and CA powders and the dry-mixed PXC polymer binder, recorded over 4000–400 cm⁻¹; spectra are baseline-corrected and vertically offset.

Table S 2-the main results of TGA on prepared polymer films of binders (PX, PXC, PXM, PXG, and PXS).

| Sample name | Weight Loss  @150 °C (%) | Weight Loss  @700 °C (%) | T_d_  (°C) |
| --- | --- | --- | --- |
| PX | 7.4 | 91.0 | 290 |
| PXC | 3.8 | 61.3 | 303 |
| PXG | 3.9 | 72.5 | 295 |
| PXS | 3.3 | 72.5 | 304 |
| PXM | 4.5 | 72.9 | 302 |


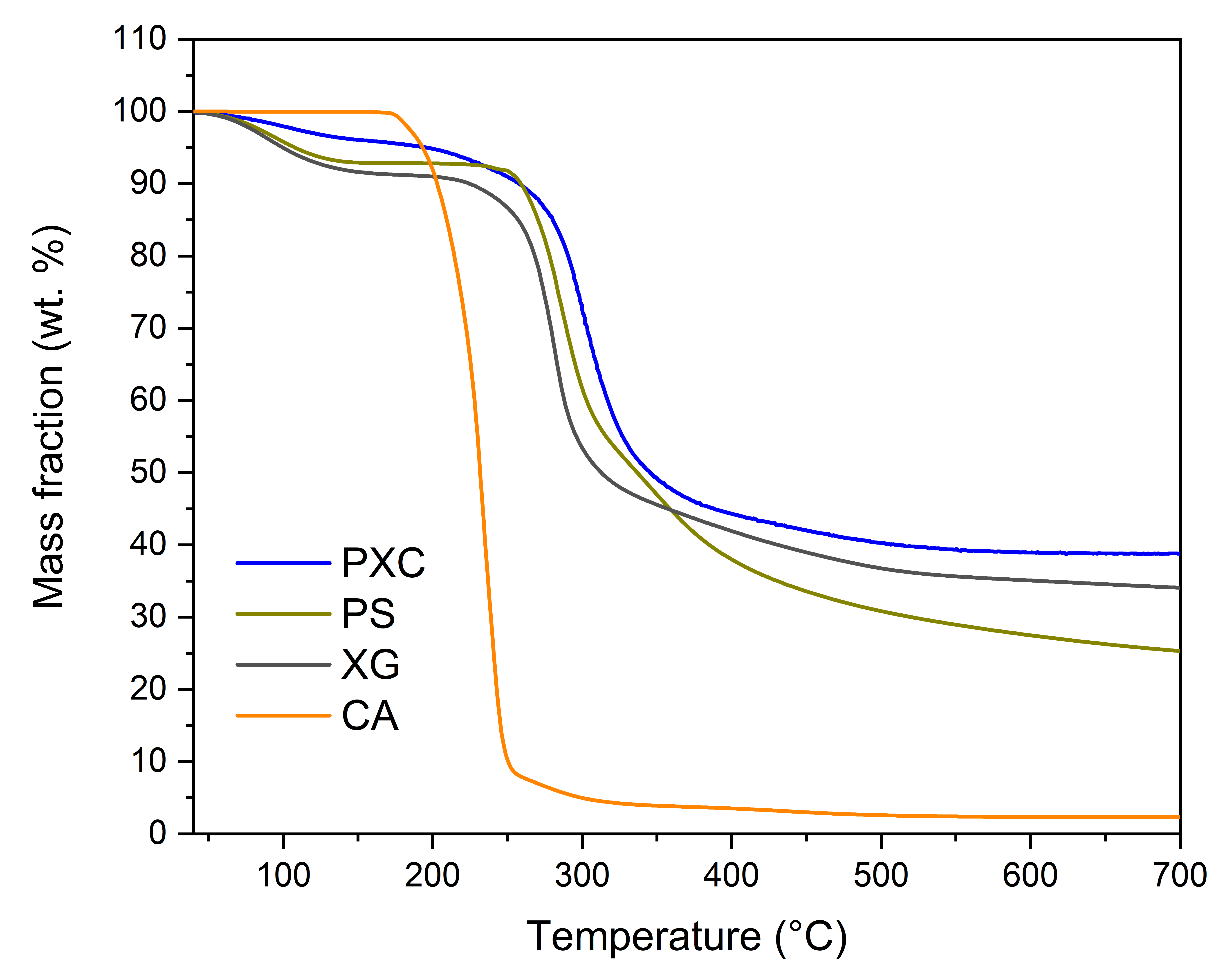


Figure S2 - TGA curves of PS, XG, CA, and PXC (cross-linked with CA), showing mass loss vs. temperature.


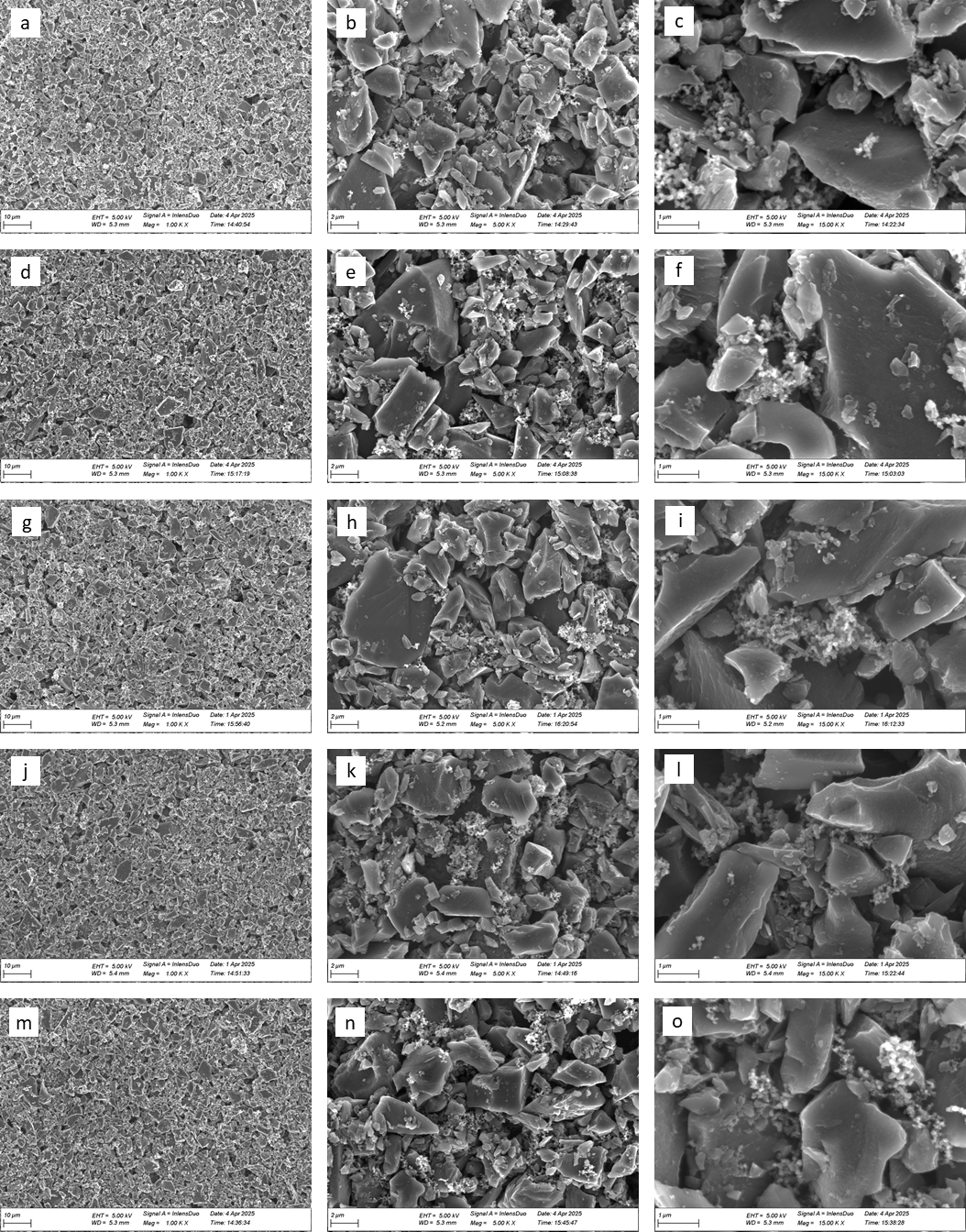


Figure S3- the SEM surface micrographs of electrodes with crosslinked and non-crosslinked binder at three magnification levels. (a, b, c) electrode with PX binder; (d, e, f) electrode with PXC binder; (g, h, i) electrode with PXM binder; (j, k, l) electrode with PXG binder; (m, n, o) electrode with PXS binder.


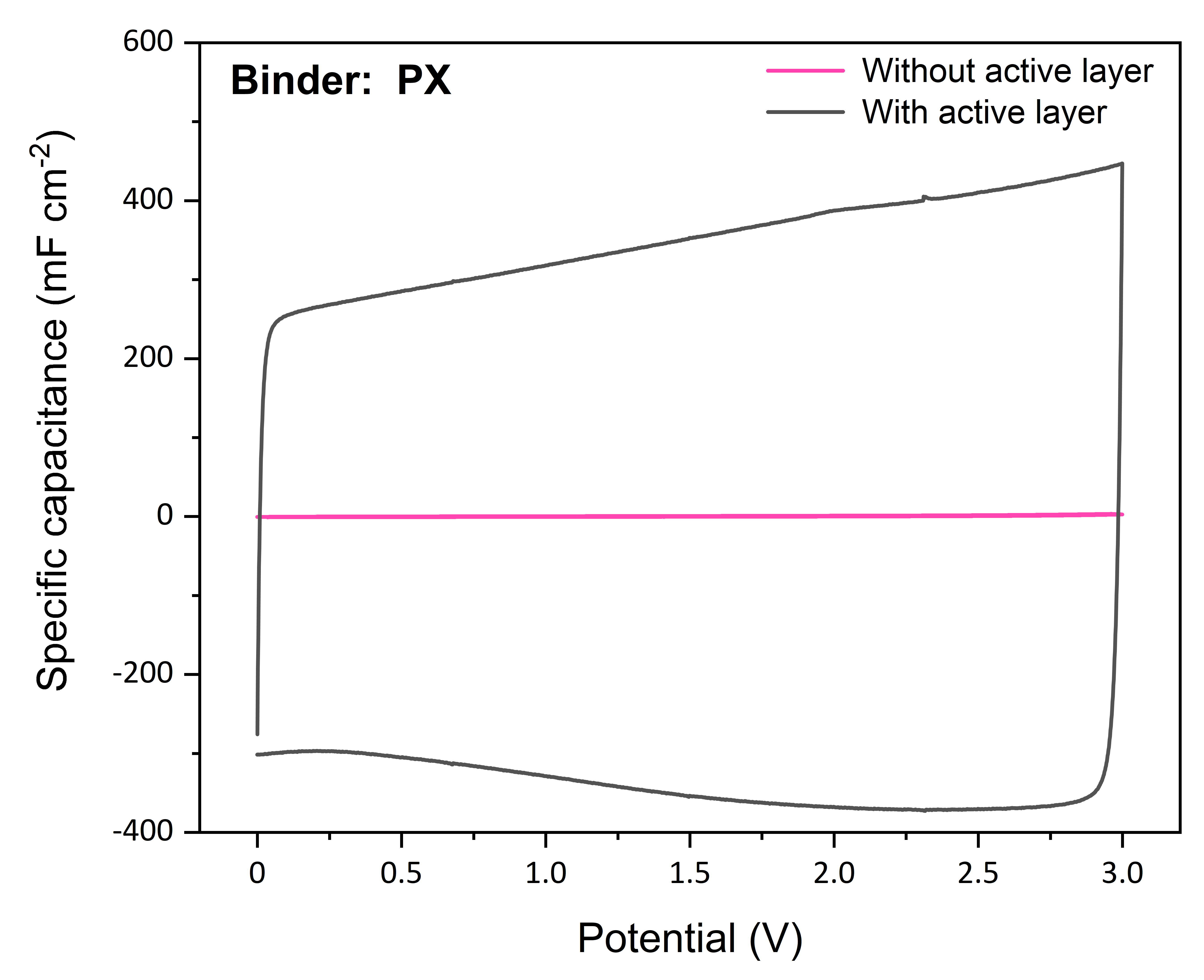


Figure S4 - CV measurements of EDLC featuring PX electrodes with and without the active layer (i.e. only primer layer). It is clear that the primer layer itself does not affect the overall capacitance.

The intrinsic electrochemical stability of the binders without any influence from the active material, we carried out additional CV measurements in a three-electrode setup, using Whatman GF/D separators, soaked with 250 µL of electrolyte to make sure the separator was completely wetted and the ion transport was stable during the measurement. Oversized activated carbon electrodes were used as counter and reference electrodes. The working electrodes were prepared with only etched Al foil, etched Al coated with PX binder, and etched Al coated with PXC binder. The CV tests were recorded at 0.5 mV s⁻¹ for 10 cycles.

The bare Al electrode shows the expected anodic oxidation peak from Al dissolution, which decreases after a few cycles due to surface passivation. For the current collector coated with PX and PXC, the same dissolution signal is visible, but the onset appears at higher potentials and the anodic current drops faster with cycling. This indicates that the binder layer helps the Al surface to passivate more quickly. Most importantly, we do not see any additional peaks or signs of binder degradation. The CV profiles stay smooth and stable across the whole potential window.


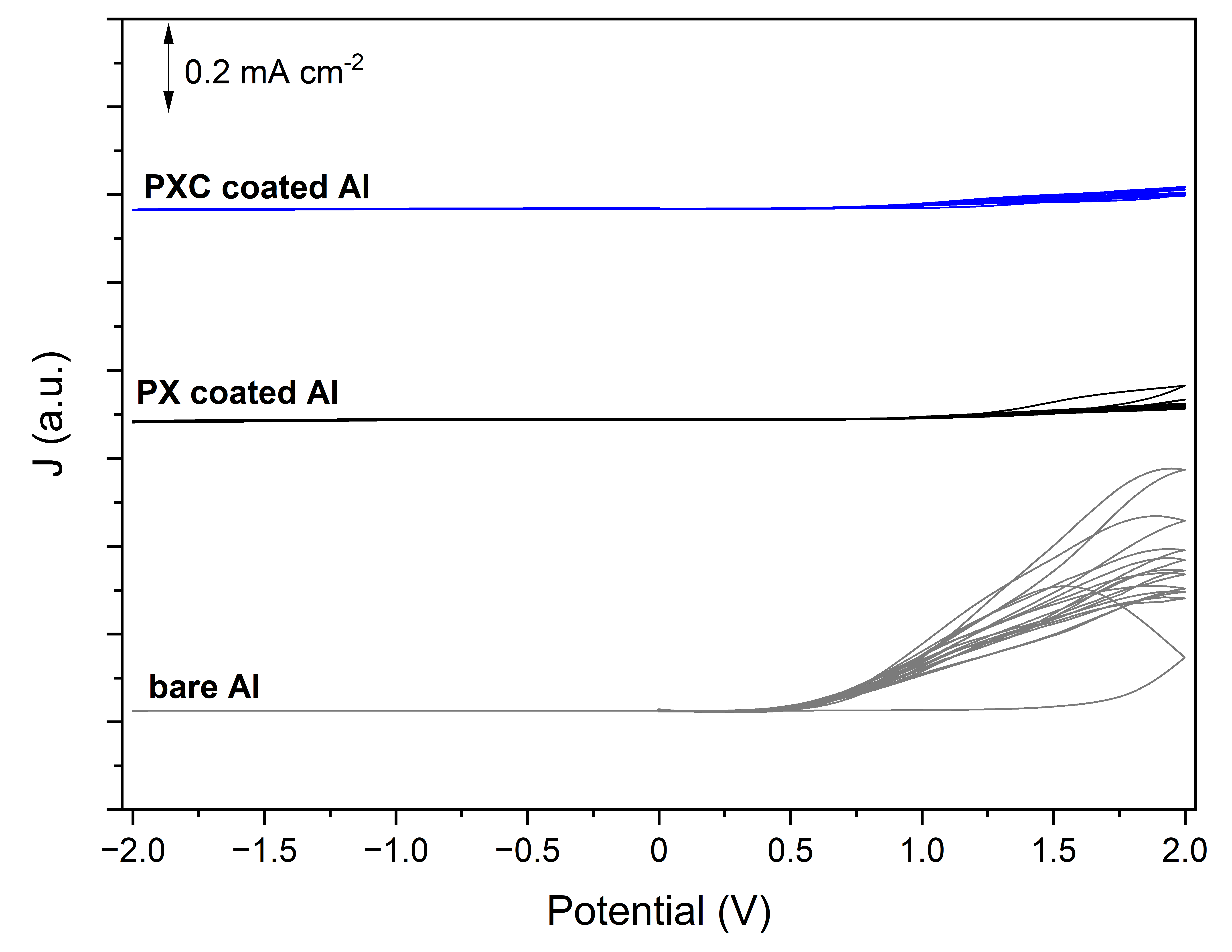


Figure S5- CV of bare Al, PX-coated Al, and PXC-coated Al electrodes at 0.5 mV s⁻¹ for 10 cycles.


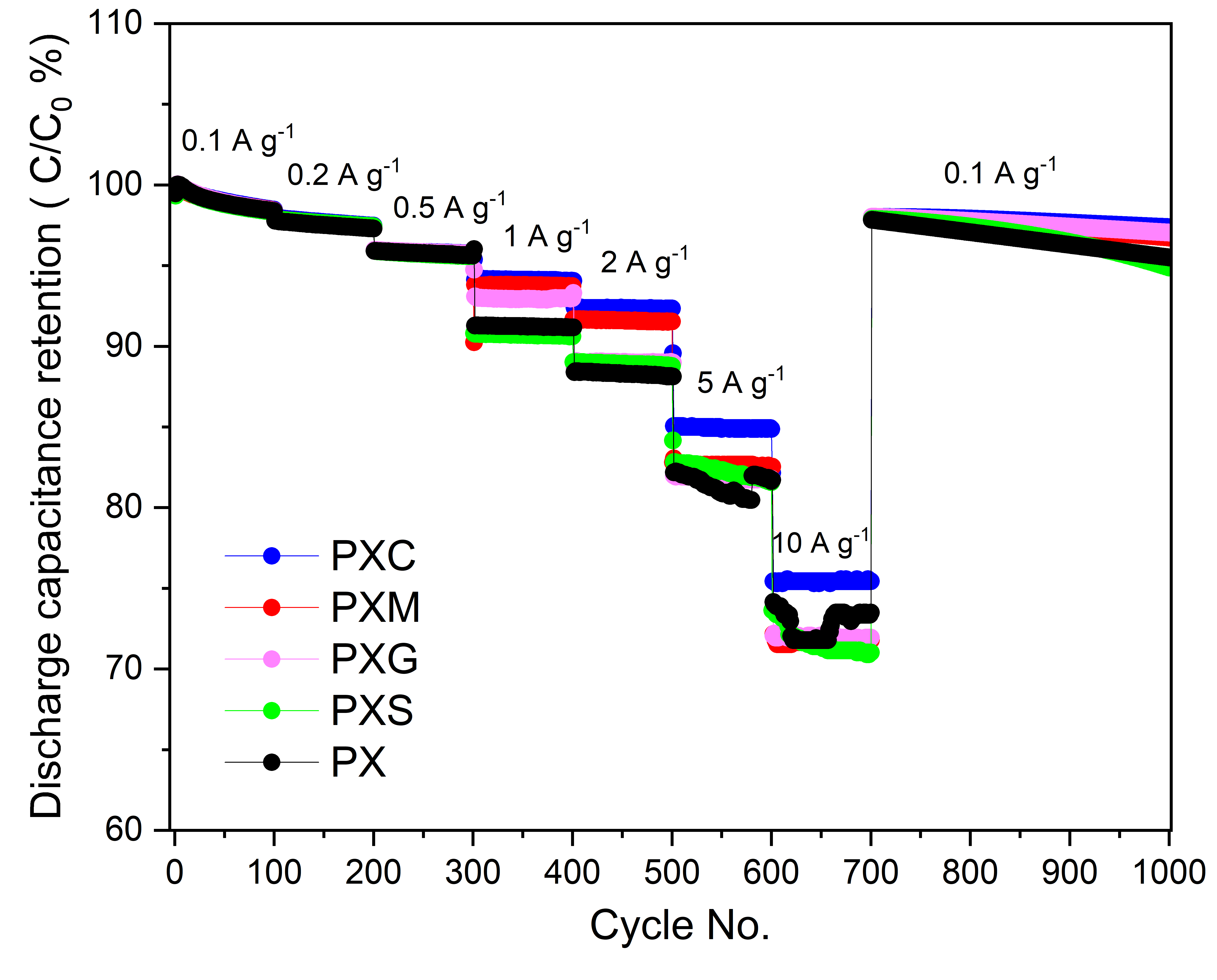


*Figure S6- Long-term cycling performance of EDLC cells using PX, PXC, PXM, PXS, and PXG binders.*

To further deepen the understanding of binder cross-linking effects, additional EIS analysis was conducted on EDLC cells employing PXM, PXG, and PXS binders, as shown in **Figure S5** b-d, respectively. These spectra were also collected within the frequency range of 1 MHz to 10 mHz, both before and after the floating voltage test. The critical parameters obtained from the fitted equivalent circuit model are summarized in **Table S2**.

In the fresh state, all three electrodes also show only one visible semicircle in the high-frequency region. Similar to PX and PXC, this is because the contact resistance (R_C_) and the adsorption/desorption process (R_ads_/C_ads_) have very close relaxation times and overlap, so their responses merge into a single depressed semicircle. This is supported by the small fitted values of R_C_ and R_ads_ for these binders. The PXM binder cell exhibited a R_e_ of 1.22 Ω, a contact resistance (R_c_) of 1.44 Ω, and an adsorption resistance (R_ads_) of 0.17 Ω. PXG-based electrodes showed the lowest initial Re of 0.76 Ω and R_c_ of 0.78 Ω, but a slightly higher R_ads_ of 0.32 Ω. The PXS cell displayed similar R_c_ (1.44 Ω) and Re (1.26 Ω) to PXM, but the lowest R_ads_ among all (0.11 Ω). These results reflect how different cross-linking agents influence electrode performance.

After the floating voltage test, impedance increased in all cells, but the extent varied depending on the cross-linker. In the PXM electrode, R_e_ increased to 1.60 Ω (+0.38 Ω), R_c_ to 1.81 Ω, and R_ads_ slightly to 0.18 Ω, indicating stable interfacial properties. PXG, on the other hand, showed a modest rise in R_e_ to 0.85 Ω (+0.09 Ω), and R_c_ to 1.18 Ω, but a more pronounced increase in R_ads_ to 0.85 Ω, pointing to some ion transport hindrance. The PXS binder, while initially promising, showed a larger R_e_ shift to 1.51 Ω, a small increase in R_c_ to 1.66 Ω, but a significant rise in R_ads_ to 0.81 Ω, indicating that its rigid structure may have led to micro-cracking or pore clogging under voltage stress. These variations suggest that binder flexibility, network density, and cross-linker chain length all impact the long-term electrochemical behavior.


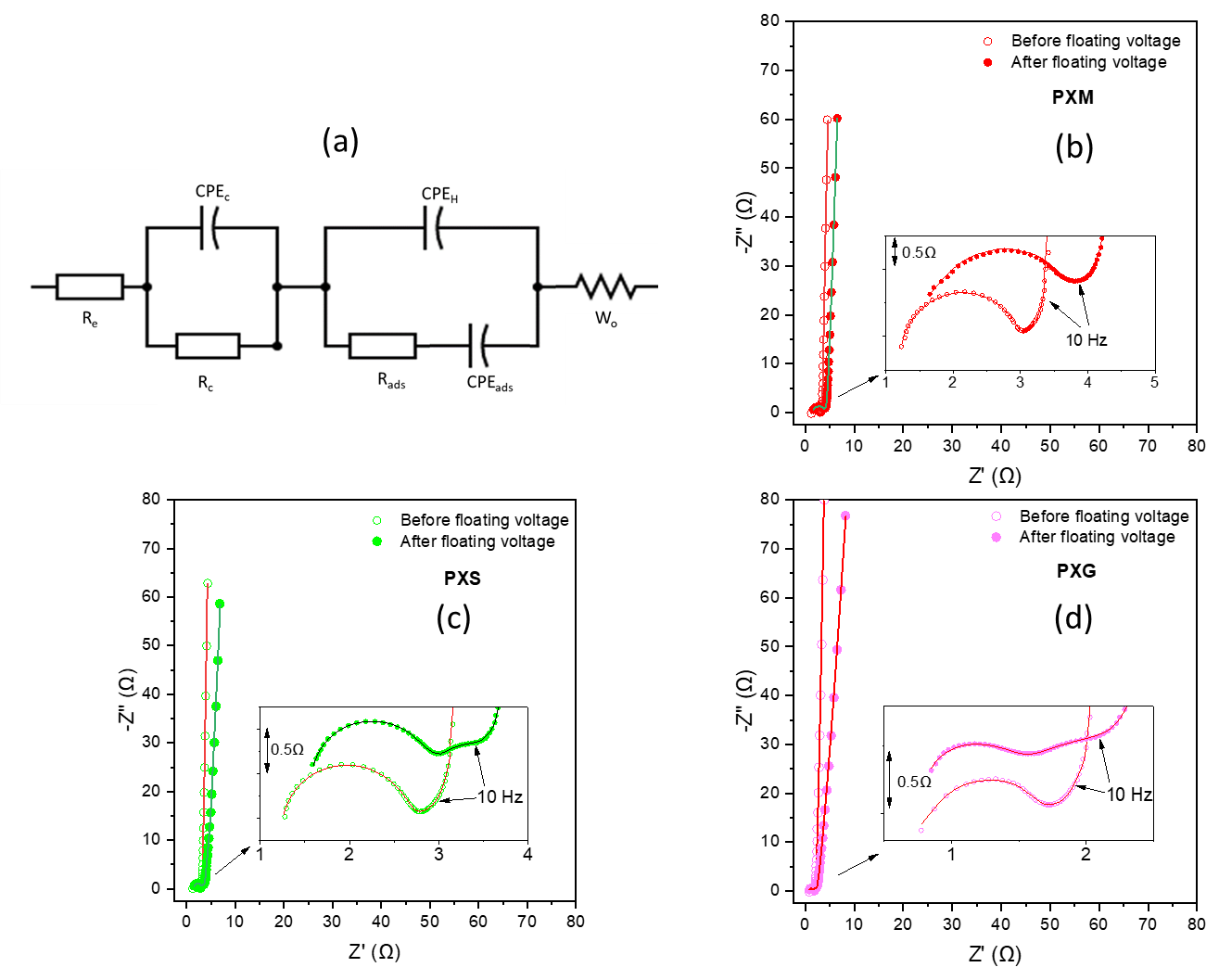


Figure S7-(a) Equivalent circuit model used to fit the EIS spectra. (b–d) Nyquist impedance plots of EDLC cells with PXM, PXS and PXG binders, recorded before and after the floating voltage test.

*Table S2- Parameters obtained by fitting EIS spectra of ELDC based on electrodes with different binders.*

| **Sample** | **Sample** | **R_e_(Ω)** | **R_c_(Ω)** | **R_ads_(Ω)** |
| --- | --- | --- | --- | --- |
| PX | fresh | 0.84 | 1.33 | 1.48 |
|  | after floating | 0.88 | 3.03 | 4.84 |
| PXC | fresh | 1.18 | 0.75 | 0.57 |
|  | after floating | 1.28 | 1.02 | 0.76 |
| PXM | fresh | 1.22 | 1.44 | 0.17 |
|  | after floating | 1.60 | 1.81 | 0.18 |
| PXG | fresh | 0.76 | 0.78 | 0.32 |
|  | after floating | 0.85 | 1.18 | 0.75 |
| PXS | fresh | 1.26 | 1.44 | 0.11 |
|  | after floating | 1.51 | 1.66 | 0.81 |
